# Natural killer cell therapy for hematologic malignancies: successes, challenges, and the future

**DOI:** 10.1186/s13287-021-02277-x

**Published:** 2021-03-25

**Authors:** Margaret G. Lamb, Hemalatha G. Rangarajan, Brian P. Tullius, Dean A. Lee

**Affiliations:** 1 Division of Hematology, Oncology, and Bone Marrow Transplant, Nationwide Children’s Hospital, 700 Children’s Drive, Suite 5A.1, Columbus, OH 43205-2664 USA; 2 Department of Pediatrics, The Ohio State University School of Medicine, Columbus, OH USA

**Keywords:** Natural killer cells, Hematologic malignancy, Cellular therapy

## Abstract

The adoptive transfer of natural killer (NK) cells is an emerging therapy in the field of immuno-oncology. In the last 3 decades, NK cells have been utilized to harness the anti-tumor immune response in a wide range of malignancies, most notably with early evidence of efficacy in hematologic malignancies. NK cells are dysfunctional in patients with hematologic malignancies, and their number and function are further impaired by chemotherapy, radiation, and immunosuppressants used in initial therapy and hematopoietic stem cell transplantation. Restoring this innate immune deficit may lead to improved therapeutic outcomes. NK cell adoptive transfer has proven to be a safe in these settings, even in the setting of HLA mismatch, and a deeper understanding of NK cell biology and optimized expansion techniques have improved scalability and therapeutic efficacy. Here, we review the use of NK cell therapy in hematologic malignancies and discuss strategies to further improve the efficacy of NK cells against these diseases.

## Background

Natural killer (NK) cells are cytotoxic lymphocytes that play a key role in recognizing malignant and virus infected cells and serve as a bridge between the innate and adaptive immune response. In hematologic malignancies, there is a qualitative and quantitative dysfunction of innate NK cells and defective NK cells at diagnosis portends a poor prognosis [1, 2]. For example, NK cell phenotypes at diagnosis of acute myeloid leukemia (AML) can be stratified into highly functional and dysfunctional groups with distinct transcriptional modifications in pathways involved in cytotoxicity, intracellular signaling, and metabolism [3]. Patients with this defective NK cell profile at diagnosis had a higher risk of relapse. In addition, patients with “hypomaturation” NK cell profiles have reduced overall and relapse-free survival [4, 5]. While the majority of clinical data supporting adoptive NK cell therapy to date is in adult myeloid malignancies, there is evidence to support the use of NK cells across a broad range of hematologic cancers, including multiple myeloma (MM). Pre-clinical data has been published for B and T cell lymphoblastic leukemia, non-Hodgkin lymphoma (NHL), and Hodgkin lymphoma (HL) [6–8]. In addition, while the graft-versus-leukemia (GVL) effect is historically thought to be more important in AML, there is mounting evidence that post-transplant immune recovery and NK cell alloreactivity confers lower risk of relapse in pediatric ALL and NHL [9–12]. Early clinical success and demonstration of safety of adoptive NK cell therapy in hematologic malignancies has led to more widespread use. As we learn more about the biology of NK cells and NK cell dysfunction in cancer, new strategies for successful NK cell therapy are emerging. This review focuses on the use of NK cell therapy to date in hematologic malignancies as well as barriers to success and future directions.

## Advantages and early success of NK cell therapy

Although the ability of NK cells to recognize and kill leukemia cells was described 45 years ago, a clinically relevant role for NK cells in the treatment of leukemia was first demonstrated nearly 20 years later [13–16]. Early NK cell recovery after stem cell transplant and increased NK cells in the graft are associated with improved transplant outcomes in leukemia [17–19]. Additional evidence of NK cell-mediated GVL was demonstrated in the setting of HLA-mismatched hematopoietic stem cell transplant (HSCT) [20, 21]. Ruggeri et al. observed that patients with AML undergoing haploidentical HSCT had decreased relapse rates when HLA differences between the donor and recipient were present in the GVL direction in a missing-ligand model for NK cells [20]. This concept was termed “ligand–ligand mismatch” and similar studies confirmed the importance of NK cell alloreactivity in AML patients undergoing HSCT [20, 22–24]. Similarly, decreased relapse and increased survival were seen in patients receiving HLA-mismatched transplants in which the donor-recipient pair was also mismatched for KIR genes [25–27].

Supported by this early clinical evidence, adoptive NK cell therapy to augment the GVL effect was investigated. The earliest trials were performed with NK cells isolated from healthy donor leukapheresis products using immunomagnetic cell selection and overnight IL-2 activation [28–30]. Using this approach, Miller et al. demonstrated that infusion of haploidentical NK cells after chemotherapy could induce remission of poor-prognosis AML [31]. In a similar study, Rubnitz et al. reported the safety of KIR-mismatched NK cell infusion as post-remission consolidation therapy for children with AML, with no relapses reported in the 10 patients treated [32]. A similar approach has been used for adoptive transfer of NK cells in patients with refractory lymphoma and MM [33, 34]. Importantly, GVHD was not reported in any of these studies utilizing allogenic NK cells. Other studies using NK cells derived by this approach in the allogeneic HSCT setting in patients with lymphoid and myeloid malignancies have also demonstrated that NK cell infusions were safe and not associated with severe infusion reactions, GVHD, or graft rejection [35–38]. However, the response rates in these studies were variable (OS from 29% to 73%) and the NK cell doses produced by this approach were typically limited to a single dose of ≤10^7^ /kg.

**Fig. 1:**
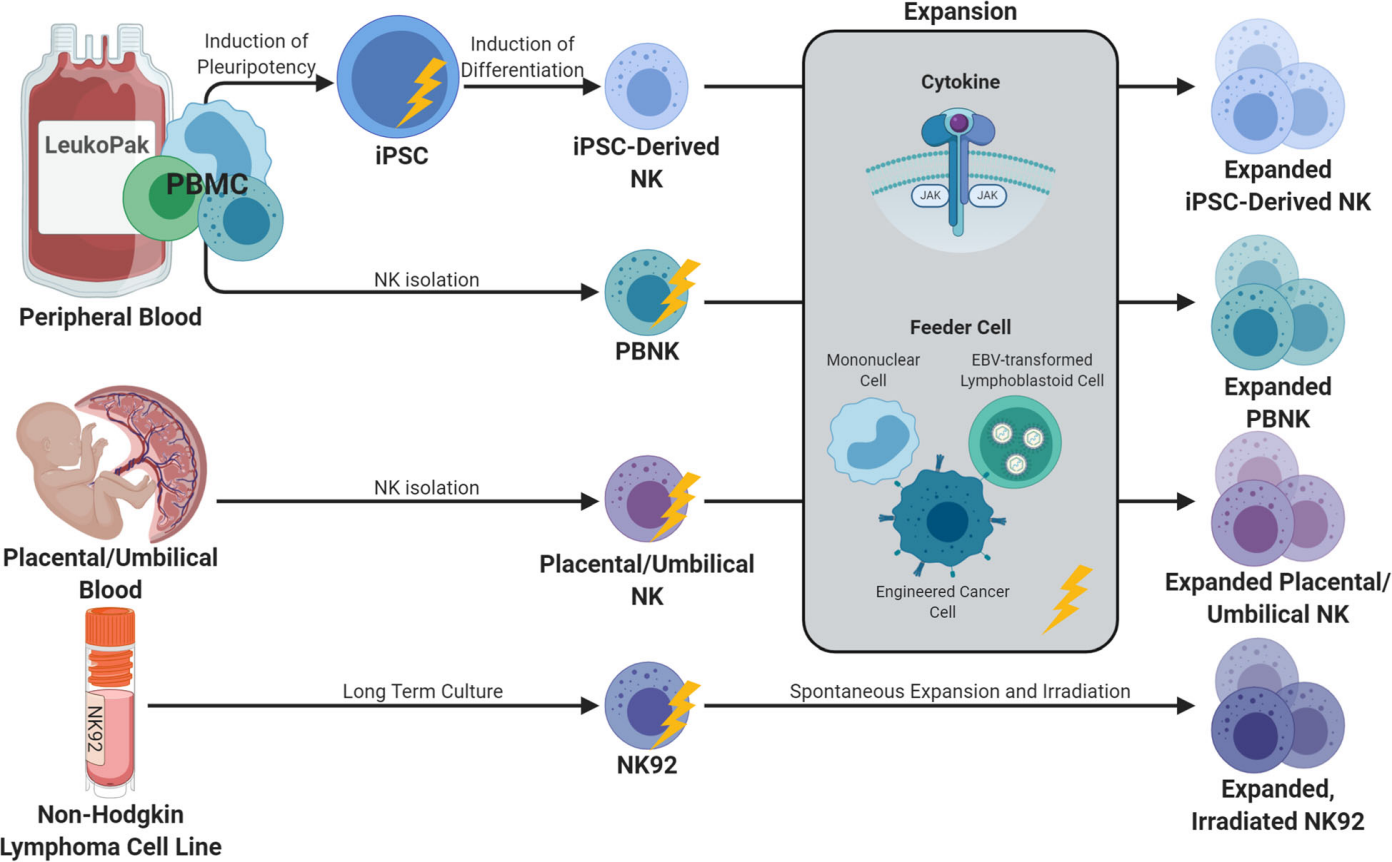
Sources for expanded NK cells. Current NK cell therapies are derived from peripheral blood (PB) NK cells, umbilical cord/placental NK cells, NK cell lines, or induced pluripotent stem cells (iPSC). Isolated NK cells are expanded utilizing cytokine stimulation with or without the presence of feeder cells. Yellow lightning bolts indicate timepoints utilized for genetic alteration of the final expanded NK cell product

Advancements in NK cell sources and expansion methods have improved the potential for NK cell therapy by enabling repeated dosing with larger numbers of NK cells (Fig. 1). Expansion methods have been developed that use cytokines alone, or in combination with costimulatory antibodies or agonists [39, 40]. The development of irradiated feeder cells generated from autologous mononuclear cells, EBV-transformed lymphoblastoid cell lines, or tumor cell lines has led to improved NK cell maturation and proliferation ex vivo [41–48]. Feeder cells have also been genetically modified to express membrane-bound cytokines and co-stimulatory molecules such as 4-1BB, MICA, IL-15, and IL-21, which can result in > 1000-fold expansion over a period of weeks. NK cell growth may plateau, however, because of exhaustion and/or proliferative senescence attributed to shortened telomeres [46, 49, 50]. In our experience with irradiated K562 feeder cells expressing 4-1BB and membrane bound IL-21, the IL-21 leads to STAT3-mediated induction of telomerase reverse transcriptase and increased telomere length. NK cells expanded with this method are highly functional and do not show proliferative senescence, achieving an average 3000-fold expansion in 2 weeks and 20–80,000-fold in 3 weeks [7, 44].

There have also been significant advances utilizing alternative allogeneic NK cell sources such as the umbilical cord blood, NK-like leukemia/lymphoma cell lines, and stem cell-derived NK cells, and there are advantages and disadvantages to each method (Fig. 1). While absolute lymphocyte numbers are low in UCB, it is relatively rich in NK cells compared to the peripheral blood, comprising 20–30% of the CB lymphocyte population [51]. The volume of the cord blood is limited, however, and freshly isolated CB NK cells are phenotypically immature and less cytotoxic with low expression of CD16 and activating receptors [51]. These quantitative and qualitative limitations of CB NK cells, however, can be overcome with cytokine stimulation and expansion, and expanded CB NK cells are highly functional against tumor targets [52–54]. A phase 1 study of cord blood-derived NK cells combined with autologous SCT in MM demonstrated the safety of NK cell doses up to 1 × 10^8^/kg with no dose-limiting toxicity, no GVHD, and NK cells detectable in the peripheral blood for up to 26 days [55].

The NK cell lymphoma cell line, NK-92, has high proliferative capacity in culture, exerts anti-tumor cytotoxicity and is easily genetically modified using viral or non-viral transduction techniques [56]. While the infusion of a malignant cell line has the theoretical potential to cause malignancy itself, irradiation of the NK-92 cells prevents proliferation in vivo and there have been clinical trials demonstrating the safely of this method [57–60]. However, this limits in vivo persistence and eliminates in vivo expansion. Finally, there has been extensive study optimizing the generation of functional NK cells from embryonic stem cells and induced pluripotent stem cells (iPSC) [61, 62]. The advantages of this method include a highly uniform, standardized, end product, and relative ease of genetic engineering of these cells [63].

Clinical trials utilizing expanded NK cells have shown a high degree of safety and early evidence of benefit in both the transplant and non-transplant settings. Haploidentical IL-21-expanded NK cells delivered in the setting of haploidentical transplant with post-transplant cyclophosphamide led to a dramatic reduction in post-transplant relapse in patients with myeloid malignancies. Compared to historical matched controls, delivery of three infusions of NK cells (on days − 2, + 7, and +28) reduced leukemia relapse from 35 to 5%, respectively [64, 65]. Expanded NK cells have also been used safely in the setting of autologous SCT for MM in combination with lenalidomide [55]. Outside of the transplant setting, expanded NK cells in combination with chemotherapy yielded a 69% complete response rate in a phase I study of patients with relapsed/refractory AML [66]. In these early clinical trials, NK cells have been safe with no dose-limiting toxicity, cytokine release syndrome, or increase in rates of GVHD. Given these initial reports demonstrating safety and efficacy, there are currently dozens of clinical trials exploring the use of NK cells for hematologic malignancies.

## Barriers to NK cell success and solutions

### Pharmacokinetics: expansion and persistence of NK cells in vivo

In contrast to T and B cells that can persist for months to years, innate immune cells are believed to be relatively short-lived, lasting for days to weeks [67]. In addition to the already finite lifespan of NK cells, donor NK cells face the additional challenge of allo-rejection. The importance of cell persistence for the success of CAR T cell therapy in leukemia is well described [68]. Similarly, in vivo expansion and persistence of NK cells after adoptive transfer is likely to be important for clinical efficacy. Grzywacz et al. demonstrated that increased NK cell homing and persistence in the bone marrow 2–3 weeks after NK cell infusion for relapsed AML was important for clinical response [69]. Strategies to improve persistence and in vivo expansion of NK cells after adoptive transfer include pre-infusion lymphodepleting therapy, co-administration of cytokines, and repeated NK cell infusions. Recent studies revealing the ability of NK cells to develop immunological memory clearly demonstrate that the function and persistence of NK cells is enhanced by tumor cell or cytokine priming [70–74]. Cytokine primed or expanded NK cells, however, may become “addicted” to cytokine stimulation and undergo apoptosis upon cytokine withdrawal [75]. This highlights the need for endogenous or exogenous cytokine stimulation after adoptive transfer to facilitate persistence.

Early clinical trials with IL-2 activated haploidentical NK cells utilized lymphodepleting chemotherapy with cyclophosphamide and fludarabine as well as subcutaneous IL-2 to facilitate NK cell expansion and persistence [31, 32]. Miller et al. demonstrated that adequate lymphodepletion with high-dose cyclophosphamide and fludarabine led to in vivo NK cell expansion with persistence of donor NK cells for over 28 days in some patients [31]. There was an inverse correlation between lymphocyte count and endogenous serum IL-15 after lymphodepleting chemotherapy suggesting that successful NK cell expansion was secondary to both clearing of alloreactive recipient cells and IL-15 stimulation of NK cells. The administration of exogenous IL-2 not only promotes NK cell priming and proliferation but also selectively targets regulatory T cell expansion and is utilized to promote immune tolerance in autoimmune disease such as type 1 diabetes [76, 77]. The immunomodulatory effect of regulatory T cell expansion in response to exogenous IL-2 in cancer is not clear, however, and it is worth noting that the adoptive transfer of NK cells alone may increase the proportion of Tregs [78]. IL-15 stimulates CD8+ T cells, has a critical role in NK cell development, and promotes NK cell survival via expression of the anti-apoptotic factor Bcl-2 [79]. In addition to its role in NK cell development, IL-15 is critical for the survival of mature NK cells in vivo [79]. Using IL-15 in lieu of IL-2 in the setting of haploidentical NK-cell therapy led to improved NK cell expansion in vivo, however, still came with the unwanted side effects of cytokine release syndrome, neurologic toxicity, and NK cell exhaustion [80]. The IL-15 super agonist, ALT-803, was designed to extend the cytokine half-life and mimic the physiologic cell to cell trans-presentation of IL-15. A phase I trial of ALT-803 in relapsed AML/MDS post-transplant led to a more robust NK cell expansion than the traditional recombinant human IL-15 and a more tolerable toxicity profile with no cytokine release syndrome, GVHD, or other dose-limiting toxicities reported [81]. Clinical efficacy as monotherapy in this setting was limited, however, with only one patient out of 27 achieving a complete response. Clinical trials utilizing ALT-803 in combination with adoptive NK cell therapy for AML are underway (NCT02890758, NCT03050216). To overcome the issues with toxicity of exogenous cytokines, others have engineered NK cells to express novel receptors to enhance proliferation and function. Hematopoietic growth factors used to stimulate the erythropoietin receptor (EPOR) or thrombopoietin receptor (c-MPL) have demonstrated clinical safety. Viral transduction of NK-92 cells to express EPOR or c-MPL led to increased NK cell survival and cytotoxicity in response to TPO or EPO ligands [82].

Another strategy for improving NK cell persistence and clinical efficacy is repeated infusions of donor NK cells. While previous studies were limited by small NK cell numbers harvested from donor the peripheral blood, newer strategies for ex vivo NK cell expansion have allowed for repeated doses of high numbers of NK cells, reducing the need for in vivo expansion. Importantly, these NK cells also have a highly functional phenotype with improved cytotoxicity and cytokine secretion against a variety of cancers. NK cells expanded with IL-21-expressing irradiated feeder cells are highly metabolically active with a memory-like NK cell phenotype and increased expression of activating receptors and chemokine receptors associated with NK cell trafficking and persistence [83]. The persistence of expanded NK cells after adoptive transfer has not been extensively studied; however, a mouse model of IL-21 expanded NK cells suggests that expanded NK cells can survive for up 21 days without the need for exogenous cytokine stimulation [84]. Adoptive transfer at day 7 post-transplant correlated with increased number, function, and phenotype at day 28, suggestive of persistence [64]. These data suggest that in addition to improved NK cell numbers, expanded NK cells have anti-leukemic efficacy and potential for in vivo expansion and persistence.

Particularly in patients with urgent medical need such as in relapsed acute leukemias, an important consideration for adoptive NK cell therapy is the fast turnaround time needed to generate the therapeutic product. In this patient population, the time it takes to work up a donor, collect, and expand NK cells may allow for the leukemia tumor burden to grow out of control. In addition, NK cells from cancer patients are low in number and function and demonstrate limited expansion, providing insufficient numbers for effective autologous therapy [85]. For these reasons, the development of an allogenic NK cell bank for “off-the-shelf” therapy is desirable. Approaches include utilizing cord blood NK cells, iPSC-derived NK cells, and unrelated “optimal” donor peripheral blood NK cells. The latter strategy identifies donors who have HLA and KIR genotypes for optimal education, a high proportion of activating KIRs, and who have been exposed to CMV resulting in NKG2C+ “memory-like” NK cells. We have developed a universal-donor NK cell bank utilizing these “optimal” donors in collaboration with Be-the-Match Biotherapies (BTMB), and expanded NK cells from this bank are being utilized in a clinical trial for relapsed AML/MDS (NCT04220684). The use of these off-the-shelf NK cell sources will become increasingly important in the development of genetically engineered NK cell programs.

### NK cell recognition of tumors: aberrant receptor/ligand expression

One advantage of NK cell therapy is the ability of NK cells to recognize tumors without the need for antigen presentation. In contrast to T cells, NK cell activation is tumor antigen-independent and is instead regulated by a balance of activating and inhibitory NK cell receptor signaling. Activating receptors recognize ligands on the surface of cancer or viral infected cells that signal danger, and inhibitory receptors are responsible for recognition of self. NK cell receptor classes include natural cytotoxicity receptors (NCR), C-type lectin receptors, and killer cell immunoglobulin-like receptors (KIRs). The presence of NK cell activating receptor stress ligands on the surface of tumor cells is crucial for NK cell recognition of these cells as abnormal. NK cell dysfunction via altered activating receptor expression or tumor downregulation of NK cell receptor ligands is a common method of tumor immune escape. For example, the absence of NKG2D and other NCR ligands on leukemic blasts allows them to escape NK cell surveillance [86, 87]. The DNAM-1 receptor/ligand axis is altered in patients with AML with both downregulation of DNAM-1 receptors on NK cells and low expression of DNAM-1 ligands (CD112/155) on AML clones leading to poor NK cell conjugation and killing [88, 89]. NK cells from patients with hematologic malignancies exhibit low expression of activating NCRs, including NKp46, DNAM-1, and NKG2D which impairs their effector function and predicts poor response to therapy [8, 90–92].

To address this, monoclonal antibodies to block inhibitory KIRs or stimulate NK cell activating receptors can tip the inhibition/activation balance in favor of NK cell activation. Inhibitory KIRs recognize HLA molecules, are distinguished by the number of extracellular immunoglobulin domains (2D or 3D), and are assigned an “L” to indicate that they have a long cytoplasmic tail containing immunoreceptor tyrosine-based inhibitory motifs (ITIMs). IPH2101 is an anti KIR2DL antibody that is being studied in AML, MM, B cell lymphoma with pre-clinical, and early clinical evidence of efficacy—particularly when combined with lenalidomide [93–95]. A follow-up phase II study in MM failed to demonstrate clinical efficacy, which may be secondary to blockade of normal NK cell education or licensing through inhibitory KIRs [96, 97]. Clinical trials utilizing fully licensed, expanded NK cells in combination with IPH2101 may elicit improved responses.

Stimulation of activating receptors is another way to tip the balance toward NK cell activation, and as mentioned above, tumors often downregulate NK cell receptor ligands to escape immune surveillance. NKG2D is a one of the most important activating receptors in the NK cell repertoire and recognizes cellular stress ligands MICA, MICB, and ULBP1-6. NKG2D also serves as a co-stimulatory receptor on cytotoxic T cells. Synthetic activation of the NKG2D receptor via NKG2D ligand and antitumor antibody fusion or NKG2D ligand/cytokine fusion is one way to overcome immune escape and facilitate NK cell-tumor interaction and may also serve a dual function in activation of T cells [98–100].

A key pathway responsible for NK cell recognition of tumors is via CD16 receptor recognition of antibody coated targets in a process called antibody dependent cellular cytotoxicity (ADCC). Binding of the crystallizable fragment (Fc) of IgG to the Fcγ receptor III (FcγRIIIa/CD16a) on NK cells creates a bridge between the NK cell and the tumor cell and leads to NK cell-mediated tumor lysis. This is an important mechanism of therapeutic efficacy for some anti-tumor monoclonal antibodies. In hematologic malignancies, rituximab (anti-CD20) and daratumumab (anti-CD38) are NK-cell-dependent antibodies that are widely utilized for B cell malignancies and MM, respectively. Combining monoclonal antibodies with adoptive NK cell therapy may further enhance tumor recognition by expanded NK cells.

It is worth noting the limitations of monoclonal antibodies and potential technologies to improve them. First, individual genetic polymorphisms of the CD16a have variable affinity to bind IgG [101, 102] and patients with the low affinity CD16 receptor may have suboptimal clinical responses to monoclonal antibody therapy [103]. In addition, the success of monoclonal antibody therapy is dependent on functional effector NK cells. With these concepts in mind, novel antibody constructs to simultaneously enhance the NK cell-tumor cell immune synapse and increase NK cell numbers are in development. Bi-specific and tri-specific killer engagers (BiKEs and TriKEs) are small molecule constructs composed of a single-chain variable fragment (scFv) (comprised of the heavy and variable light chains of an antibody connected by a short peptide) of one antibody linked to another scFv and/or cytokine. Most of the BiKEs/TriKEs in development include a high affinity anti-CD16 component to overcome the polymorphism differences in CD16 affinity mentioned above. The anti-CD16 component is combined with one or two tumor antigen-specific antibodies such as CD19/20 in B cell malignancies [104–107], CD33/CD123 in AML [108–110], CD30 in Hodgkin’s lymphoma [111], and HLA class II in lymphoma [112]. The newer generation of TriKEs incorporate cytokine stimulation to further enhance NK cell function upon antigen engagement. For example, Felices et al. engineered a TriKE that is composed of anti-CD16, anti-CD33, and an IL-15 moiety to drive NK cell activation, expansion, and persistence [113]. Finally, the role of immune checkpoints in NK cell regulation is described below and the addition of inhibitory receptor blockade/checkpoint blockade such as TGF-β inhibition to an NK cell engager may be an additional mechanism to improve NK cell ADCC [114].

Daratumumab is an FDA approved monoclonal antibody against CD38 that has changed the therapeutic landscape for MM with overall response rates of greater than 80% when combined with chemotherapy [115–117]. Pre-clinical and clinical reports have indicated that there may also be a role for daratumumab in T cell ALL and other CD19/22-negative hematologic malignancies [118, 119]. NK cells have high levels of CD38 on their surface and are depleted in patients treated with daratumumab as a result of NK-to-NK ADCC, referred to as “fratricide” [120]. CD38 negative or low NK cells are resistant to daratumumab-induced fratricide and have improved tumor cytotoxicity when combined with daratumumab compared to CD38+ NK cells [120]. To overcome NK cell fratricide induced by daratumumab, we generated CD38 knock out NK cells (CD38^KO^ NK) using CRISPR/Cas9. These CD38^KO^ NK cells are resistant to daratumumab-induced fratricide, have a superior metabolic profile, and improved ADCC against CD38 expressing MM [121]. Ongoing pre-clinical validation studies will determine if this method could be utilized across a wide range of CD38 expressing hematologic malignancies.

Combination drug therapies can increase NK cell receptor ligands on the surface of tumor cells and may be another way to improve NK cell recognition of tumors. For example, poly-ADP-ribose polymerase 1 (PARP1) plays a role in repressing expression of NKG2D ligands on AML cells and PARP inhibitors may be a therapeutic target to increase ligand expression and improve NK cell detection of leukemia stem cells [86]. In addition to improved NK cell receptor-ligand recognition, NK cells have improved cytotoxicity in combination with the PARP inhibitor olaparib against the breast, lung, and prostate carcinoma cells [122]. PARP inhibitors are also being utilized in myeloid malignancies, and their combination with adoptive NK cell therapy may further improve therapeutic efficacy.

Lenalidomide is an effective immunomodulatory treatment for MM, other B cell neoplasms, and MDS. In the context of NK cells, lenalidomide decreases the threshold for NK cell activation upon receptor stimulation, enhances antibody dependent cytotoxicity (ADCC), and upregulates receptor and ligand expression on NK cells and tumor cells, respectively [123–125]. Combination trials with lenalidomide and monoclonal antibodies have been promising in B cell lymphomas and MM, in part due to improved NK cell recognition of tumor targets via CD16 leading to ADCC [116, 126–129].

Finally, with the success of CAR T cell therapy in ALL and B cell lymphomas, there is a major push in the field to develop CAR-NK cell therapies. While T and NK cell effector functions are similar, a CAR NK cell has the added ability to recognize tumors through innate NK cell receptors, potentially preventing relapse due to antigen escape. Additionally, allogenic HLA-mismatched NK cells have been given safely without causing GVHD, highlighting the potential to produce universal donor or “off-the-shelf” CAR-NK cells to circumvent cost and timing constraints seen with manufacturing CAR-T cell therapy. Finally, NK cells are safe and cytokine release syndrome and neurologic toxicity have been minimal in NK cell trials to date. Historically, genetic modification of NK cells was unsuccessful due to NK cell resistance to viral transduction. In contrast to T cells, the innate function of NK cells as our first anti-viral defense renders them relatively resistant to traditional methods of gene modification through viral transduction. Viral transduction of peripheral blood NK cells with lentiviral vectors using a new baboon pseudo type has significantly increased high-transduction efficiency [130]. In addition, successful retroviral transduction has been demonstrated in the first phase I/II clinical trial utilizing cord blood derived CAR-NK cells, in which 11 patients with CLL or NHL were treated with a single dose of “off-the-shelf” CD19 CAR-NK cells [131]. The CAR-NK cells were equipped with CD19 CAR, IL-15, and an inducible caspase suicide gene. CAR-NK cells were well tolerated with no dose-limiting toxicity and no report of cytokine release syndrome with a response rate of 73%. CAR-NK cells expanded in vivo and were detectable for a least a year after infusion. Similar to data reported in CAR-T cell trials, patients who responded to therapy had a higher peak expansion of CAR-NK cells than those with no response. Alternative NK cell sources and newer methods of genetic engineering have enabled successful genetic modification of NK cells using non-viral methods. Non-viral transfection with electroporation and the use of transposons and CRISPR/Cas9 has led to improved efficiency and stable genomic insertion of NK cells [132]. Additional CAR NK cell targets being developed for hematologic malignancies include CD33, CD38, CD123, CD20, BCMA, CLL1, and FLT3 [58, 130, 133–138], and dual-target CARs such as CD19/20 or CD19/22.

### NK cell dysfunction within the tumor micro-environment

#### Hypoxia

Hypoxia in the TME drives angiogenesis and facilitates cancer progression and chemotherapy resistance largely through induction of the hypoxia-inducible factor (HIF) pathway as well as the PI3K/AKT/mTOR and NFκB pathways [139]. Within the bone marrow niche, pockets of hypoxia are essential for physiologic stem cell function and severe hypoxic conditions develop in the progression of hematologic malignancies including MM, leukemia, and lymphoma via similar mechanisms as solid tumors [140]. The anti-tumor immune response is dysfunctional under hypoxic conditions leading to T cell apoptosis, NK cell dysfunction, and promotion of Treg differentiation which further facilitates tumor survival [141, 142]. NK cell cytolytic function is impaired in hypoxic environments in part via decreased surface expression of activating receptors such as NKp46, NKp30, and NKG2D and CD16 [143–145]. Even after NK cell recognition of tumor cells, hypoxia-induced autophagy in cancer cells leads to degradation of granzyme B, rendering NK cells in hypoxic tumor microenvironments less cytotoxic [146].

**Fig. 2:**
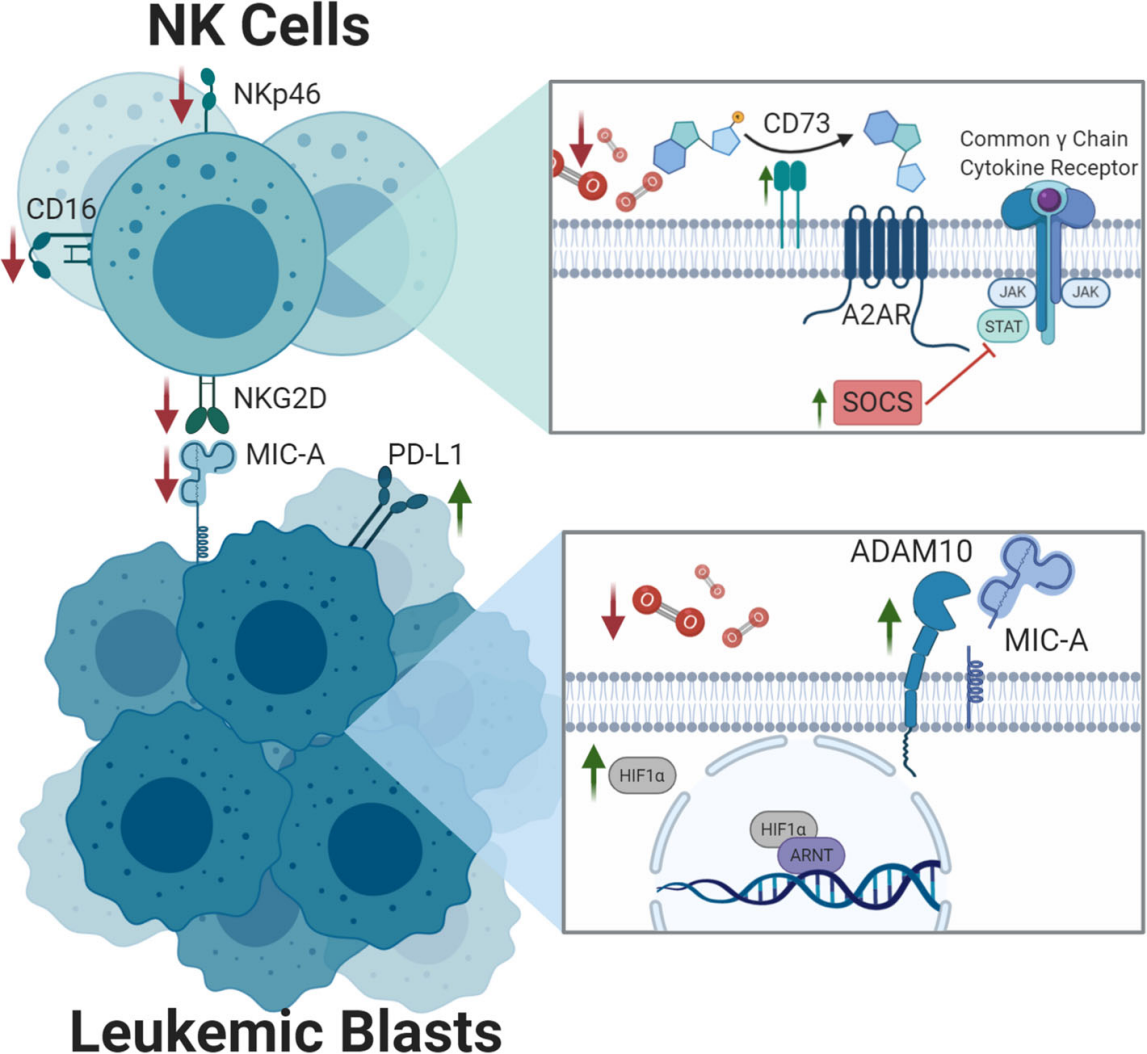
Hypoxia. Hypoxia driven upregulation of CD73 leads to increased adenosine binding at the A2A receptor (A2AR). The A2AR inhibits NK cell function through the SOCS pathway via downregulation of activating receptors. Increased expression of the hypoxia inducible factor 1-alpha (HIF1α) in leukemic blasts results in upregulation of ADAM10 and subsequent cleavage of MIC-A—the canonical ligand for the NK cell activating receptor NKG2D

NK cell dysfunction in the TME is also due to overexpression of the adenosine nucleotidase, CD73. Increased CD73 expression leads to extracellular accumulation of adenosine which has significant immunometabolic effects on NK cells [147] (Fig. 2). Adenosine stimulation of the A2A receptor on NK cells leads to negative effects on NK cell metabolism, cytokine production, and cytotoxicity [147]. A2AR blockade can restore NK cell function in the tumor environment [148] and targeting of the CD73/adenosine pathway may serve to boost the anti-tumor immune response as well as suppress tumor stem cell function [149].

In addition to intrinsic NK cell dysfunction, hypoxia can also lead to tumor cell immune evasion via upregulation of checkpoint molecules and downregulation of NK cell ligands [150, 151]. The NKG2D ligand MICA is downregulated on tumor cells in the setting of hypoxia via HIF-1α induced expression of ADAM10 [150, 152] (Fig. 2). HIFs have also been shown to induce PD-L1 expression on the surface of tumor cells via interaction with PD-L1 gene promotors [151, 153]. Therapeutic blockade of the transcription factor HIF1 or ADAM10 may overcome hypoxia-induced dysfunction of NK cells [154].

#### Checkpoints (PD-1, TIM-3, TIGIT)

**Fig. 3:**
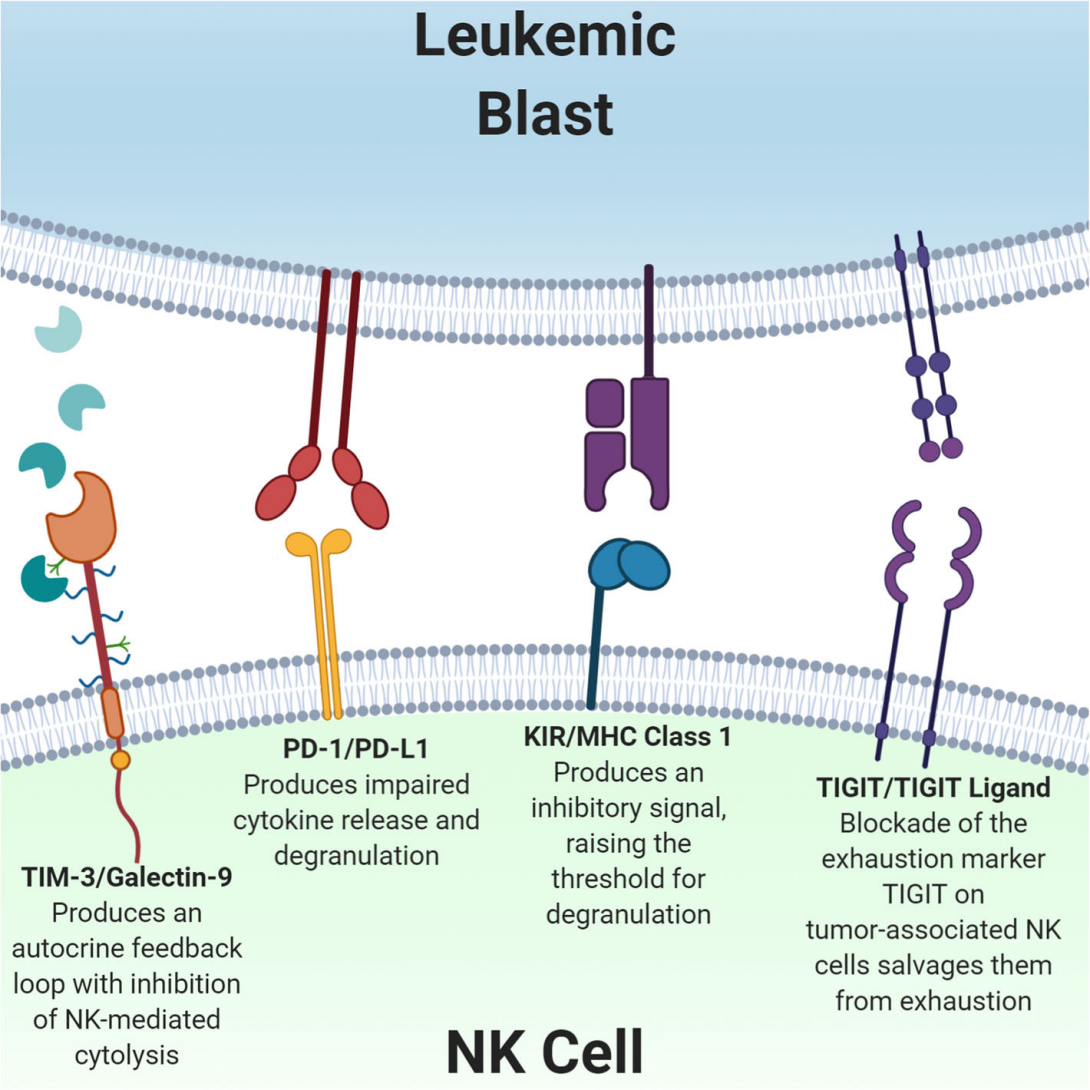
Immune checkpoints. Binding of leukemic cell-secreted galectin-9 at TIM-3, PD-L1 at PD-1, or TIGIT ligand at the TIGIT receptor inhibits NK cell cytotoxicity. Similarly, tumor cell expression of MHC Class 1 molecules leads to NK cell tolerance through interaction with inhibitory KIRs. Blockade of these immune checkpoint pathways reverses NK cell dysfunction in the tumor microenvironment

In addition to HLA class I-specific inhibitory receptors, NK cells express traditional immune checkpoint molecules such as PD-1, TIM-3, and TIGIT [155] (Fig. 3). While TIM-3 and TIGIT are expressed in healthy donor NK cells and are likely involved as conventional checkpoints for NK cells, the role of traditional T cell checkpoints like PD-1 in NK cell immune tolerance is not as well defined [156].

TIM-3 is constitutively expressed on NK cells from both healthy donors and cancer patients, and high expression of TIM-3 on NK cells has prognostic significance in solid tumors [157, 158]. In hematologic malignancies, Galactin-9 is overexpressed by tumor cells and the Tim-3/galactin-9 autocrine loop can lead to leukemic progression conferring an additional selective advantage to tumor expression of TIM-3 ligands [159]. High levels of Galectin-9 were seen in plasma of patients with AML and Galectin-9 secreted by AML blasts impairs NK cell killing of leukemic cells in culture [160]. Blockade of TIM-3 on NK cells isolated from cancer patients reversed tumor-associated NK cell exhaustion and restores cytolytic function [161].

PD-1 is only variably expressed on subsets of healthy donor NK cells but appears to be upregulated in tumor-associated NK cells [162]. Similar to its effect on T cells, stimulation of PD-1 on NK cells leads to impaired cytokine release and degranulation that may be secondary to impaired lytic granule polarization [155]. In HL and DLBCL, PD-1^+^ is elevated in both circulating and intra-tumoral NK cells compared to healthy controls [163]. In vitro PD-1 blockade with pembrolizumab enhances NK cell cytotoxicity via direct blockade of PD-1 on NK cells and indirectly by PD-1 inhibition of NK cell suppressive tumor-associated macrophages [163]. In addition, PD-L1 blockade may actually directly enhance PD-L1^+^ NK cell function and provide anti-tumor efficacy even in PD-L1 negative tumors [164]. Dong et al. demonstrated enhanced degranulation and cytokine production from PD-L1^+^ NK cells treated with an anti-PD-L1 antibody. PD-1/PD-L1 blockade is also being studied in AML, and multiple studies have reported high expression of PD-L1 on AML blasts [164]. While early clinical trials have hinted at efficacy of checkpoint blockade in AML/MDS, the role of NK cells in clinical responses to checkpoint blockade has yet to be defined [165, 166].

The co-inhibitory receptor T cell immunoglobulin and ITIM domain (TIGIT) is also expressed on NK cells [167]. NK cells isolated from patients with NHL have high expression of TIGIT compared to healthy controls [168]. Zhang et al. found high TIGIT expression on tumor infiltrating NK cells that exhibited an exhausted phenotype with impaired cytokine secretion and degranulation [169]. NK-cell specific deficiency of TIGIT prevented tumor metastasis and improved survival in a mouse model of melanoma. Blockade of TIGIT in vivo reversed the exhaustion of tumor infiltrating NK cells and slowed tumor growth, even in a T cell deficient SCID mouse model. Importantly, the therapeutic effects of anti-TIGIT therapy depended on the presence of NK cells. In a pre-clinical model of autologous stem cell transplant for MM, TIGIT blockade significantly prolonged disease control after transplant [170] and is currently being utilized in this clinical setting (NCT04150965).

#### Indoleamine 2,3-dioxygenase

**Fig. 4:**
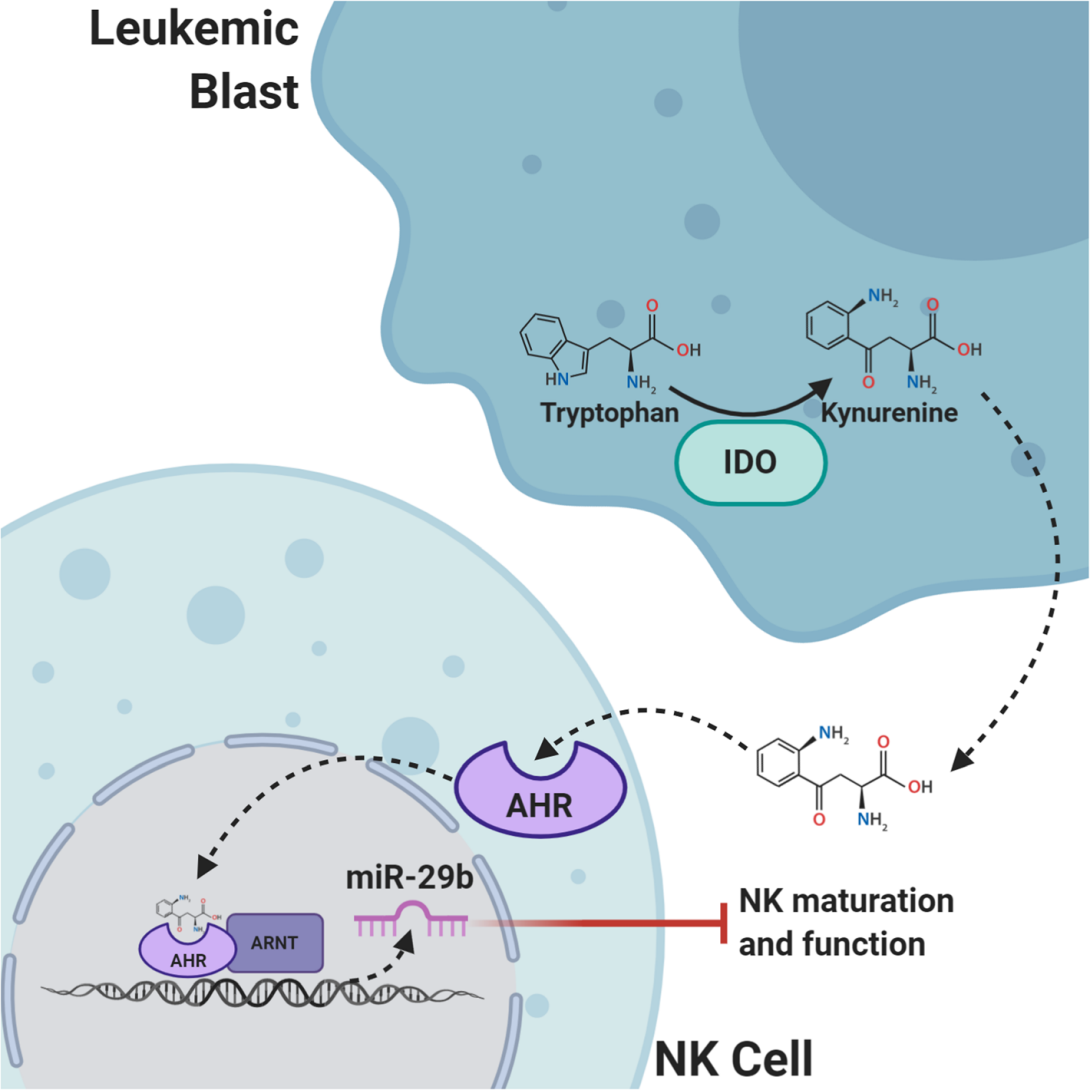
IDO. The intracellular enzyme Indoleamine 2,3-dioxygenase (IDO) catalyzes tryptophan to kynurenine, a ligand for the aryl hydrocarbon receptor (AHR). Upon kynurenine binding, AHR crosses the nuclear membrane and associates with the AHR nuclear translocator protein (ARNT). The AHR-ARNT complex binds to DNA promotor regions leading to differential expression of genes associated with immune tolerance of malignancy. In NK cells, AHR-ARNT activates transcription of miR-29b and inhibits NK cell maturation and function

Indoleamine 2,3-dioxygenase (IDO) and tryptophan 2,3-dioxygenase (TDO) are intracellular enzymes responsible for tryptophan breakdown to kynurenine. Through stimulation of the aryl hydrocarbon receptor (AhR), the IDO/TDO/Kynurenine pathway promotes immune tolerance in the tumor microenvironment via suppression of NK cells and cytotoxic T cells and promotion of regulatory T cells [171] (Fig. 4). The role of tryptophan catabolism in cancer development and progression is an active area of research; however, it is clear that IDO is overexpressed in many different cancer types, including hematologic malignancies [172]. Functional IDO is overexpressed by AML blasts which correlates with increased regulatory T cells and predicts a poor prognosis [172–174]. IDO is also overexpressed Hodgkin and NHL and IDO expression in tumor tissue of DLBCL can stratify patients at risk for chemotherapy resistance and decreased survival [175–179]. In Hodgkin lymphoma, macrophages, dendritic cells, and endothelial cells in the TME express IDO and high IDO expression is associated with high-risk features with 5-year overall survival of 67.8% compared to patients with low IDO expression who have an OS of 91.7% [178]. Karihtala et al. found that although the percentage of tumor associated macrophages expressing IDO in HL samples was low, and high IDO expression was an independent poor prognostic factor [179]. Targeting the IDO/TDO/Kyurenin pathway is of clinical interest given the broad implication in tumor development and IDO/TDO inhibitors are actively being studied in the clinic. Selective IDO inhibitors, however, fail to prevent tryptophan metabolism by the TDO pathway and vice versa so targeting the downstream AHR pathway may be more efficient [180]. In AML, upregulation of AHR ligands by blasts hinders NK cell development and function via the micro-RNA, miR-29a/b1 [181]. The use of AHR antagonists has the potential to restore NK cell development and improve NK cell killing of AML blasts.

#### Transforming growth factor-Beta

**Fig. 5:**
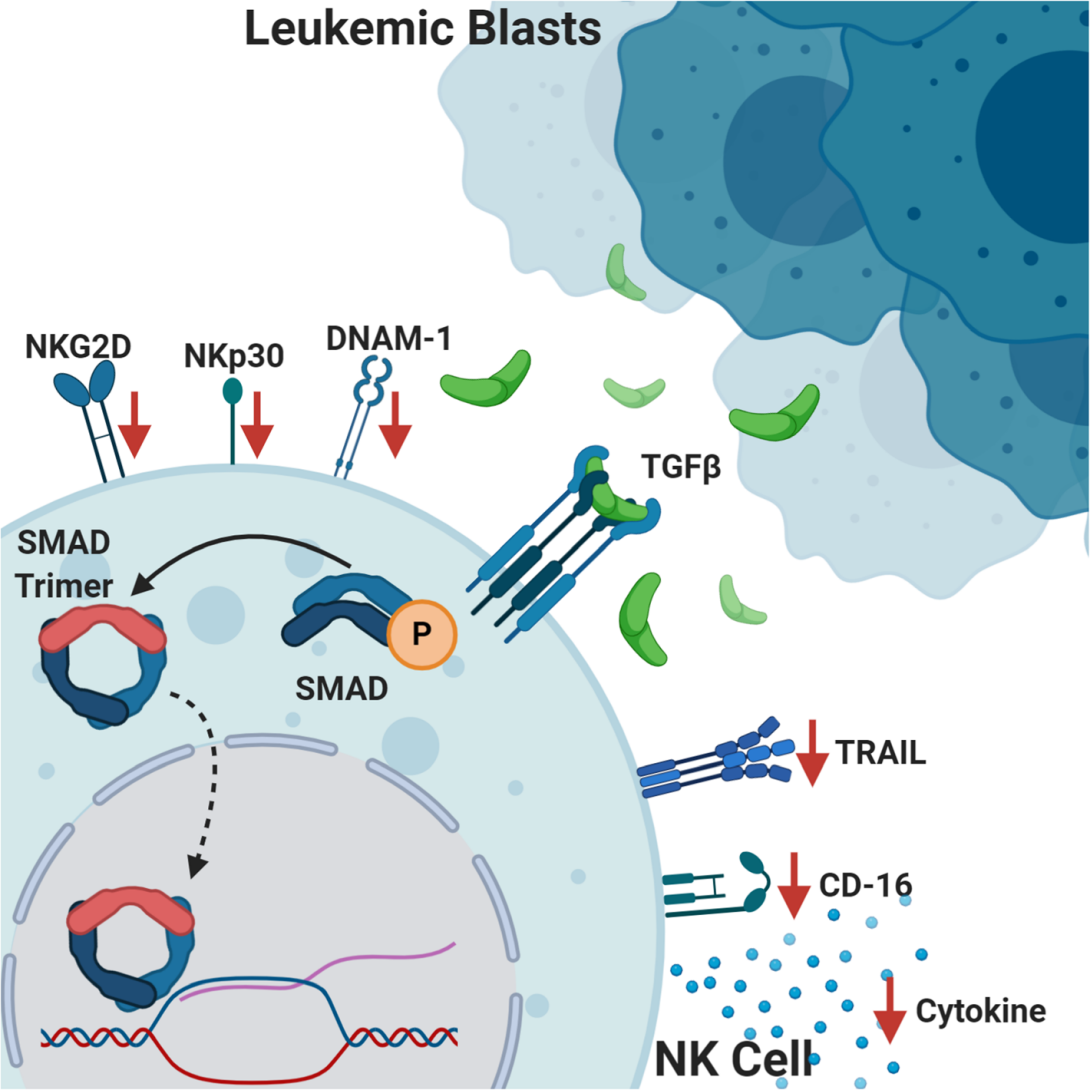
TGF-β. TGF-β secreted by leukemic blasts binds to the TGF-β receptor on NK cells leading to phosphorylation, trimerization, and translocation of SMAD complexes across the nuclear membrane. The downstream epigenetic modifications inhibit NK cell function with decreased cytokine production, impaired cytotoxicity, and downregulation of NK cell activating receptors (NKG2D, NKp30, DNAM-1, TRAIL, and CD16)

A key contributor to immunosuppression within the tumor microenvironment is transforming growth factor-beta (TGF-β) secreted by tumor cells and tumor-associated macrophages. The TGF-β family signaling pathway exerts diverse biological effects depending on the cell type and physiologic context and includes effects on cell proliferation, differentiation, communication, metabolism, and apoptosis. Within the context of cancer, TGF-β can have pro- and anti-tumorigenic effects functioning both as a tumor suppressor in pre-malignant cells and as a tumor promotor of cancer cell growth and metastasis [182]. In addition to direct effects on malignant cells, TGF-β acts as an immunosuppressive cytokine that inhibits T, B, and NK cell function [182]. Specifically, TGF-β-induced phosphorylation of SMAD2/SMAD3 in NK cells leads to decreased IFNγ production [183] and decreased anti-tumor cytotoxicity with phenotypic downregulation of the activating receptors NKG2D, NKp30, DNAM-1, TRAIL, and CD16 [184–188] (Fig. 5). The effect of TGF-β driven immune escape is well studied in pediatric and adult solid tumors [189–197]. In hematologic malignancies, aberrant TGF-β-SMAD signaling is implicated in ineffective hematopoiesis and leukemogenesis and evidence of TGF-β-mediated immune escape in leukemia/lymphoma is emerging [198–200].

Huang et al. investigated the effect of TGF-β on NK cell targeting of leukemia cells in vitro*.* The authors found that TGF-β significantly decreased NK cell killing of leukemia cell lines secondary to leukemia cell downregulation of the NK cell ligand CD48 as well as decreased ICAM-1 binding affinity resulting in impaired effector-target interaction [201]. In pediatric B-ALL, NK cell number and cytolytic function are significantly reduced at diagnosis compared to healthy controls [202]. Rouce et al. demonstrated that NK cells from the leukemia patients had an abnormal phenotype with increased expression of the inhibitory receptor NKG2A and reduced expression of the activating receptor NKp46 [202]. The authors found increased levels of TGF-β in the supernatants of ALL blast cultures and showed significant elevation in SMAD2/3 phosphorylation in NK cells isolated from patients with leukemia as well as NK cells co-cultured with ALL blasts. Importantly, blockade of TGF-β partially corrected the ALL-induced NK cell dysfunction highlighting a potential therapeutic target. In AML, defective NK cells at diagnosis are associated with increased risk of relapse and transcriptional analysis shows differential expression of TGF-β signaling pathways between highly functional NK cells and dysfunctional NK cells from patients with leukemia [3]. Decreased number and function of NK cells in the TME of lymphoma predicts a poor prognosis [203]. Similar to other solid tumors, TGF-β is expressed at high levels by both lymphoma cells and regulatory T cells within the lymphoma tumor microenvironment which is likely one of the immune escape mechanisms employed by lymphomas [204, 205]. Taken together, it is clear that similar to solid tumors, TGF-β plays a substantial role in tumor progression and immune evasion in hematologic malignancies.

Therapeutic antibodies and small molecule inhibitors targeting the TGF-β pathway are in development, but progress has been slow and focused primarily on solid tumors such as glioblastoma, pancreatic cancer, NSCLC, and hepatocellular carcinoma [206]. Galunisertib is a first in class oral inhibitor of the TGF-β receptor type 1 kinase that has shown some clinical efficacy as monotherapy or in combination with standard of care therapies [206]. In a phase II/III trial using galunisertib in low-intermediate risk MDS, 32% of transfusion-dependent patients had hematologic improvement with an acceptable safety profile. Interestingly, > 90% of patients had a > 20% reduction in plasma TGF-β levels and the authors found an increase in NK cell numbers during treatment with galunisertib. The use of galunisertib in combination with ex vivo expanded NK cells with antibody therapy reversed the TGF-β-induced suppression of cytotoxicity and led to reduction of tumor growth and improved survival in patient-derived xenografts of neuroblastoma [207]. Similar combination therapies utilizing TGF-β pathway inhibition combined with adoptive NK cell therapy have not been utilized in clinical trials to date but may enhance NK cell function in vivo.

In addition to direct TGF-β receptor blockade, NK cell engineering strategies have been utilized to overcome TGF-β inhibition of NK cells including TGF-β receptor knock out, the addition of dominant negative TGF-β receptors, and a TGF-β chimeric receptor with an intracellular NK cell activating domain [208–210]. Our lab utilized DNA-free Cas9 RNP editing of peripheral blood NK cells to successfully knock out the TGF-β receptor rendering them resistant to TGF-β-mediated suppression [208]. Yvon et al. genetically engineered cord blood NK cells using retroviral transduction to insert a dominant negative TGF-β receptor (DNRII) [210]. These DNRII-expressing NK cells exhibited normal expansion with irradiated feeder cells and had improved cytotoxicity of glioblastoma cells compared to non-transduced NK cells when exposed to TGF-β. Utilizing the same genetic modification platform, NK cells were engineered to express TGF-β receptors coupled with intracellular NK cell-specific activating domains to take advantage of receptor stimulation by TGF-β in the TME. The conversion of an inhibitory signal to an activating signal not only made these NK cells resistant to TGF-β but also led to increased NK cell activation and improved tumor control in a model of TGF-β secreting neuroblastoma [209].

Finally, our lab developed a novel platform using TGF-β stimulation during expansion with IL-2 and irradiated feeder cells. Addition of TGF-β during expansion (TGF-β imprinting) does not affect their proliferation and paradoxically and results in hyperinflammatory NK cells that produce large amounts of IFNγ, TNFα, and GM-CSF when co-cultured with tumor cells even in the presence of TGF-β [211].

## Conclusion and future perspectives

Cellular immunotherapy is on the forefront of progress in cancer research. The success of CD19 CAR T cells provided momentum to develop novel cell therapy strategies in both hematologic malignancies and solid tumors. NK cells provide an alternative cell source with a similar effector function but with decreased toxicity and the potential for an “off-the-shelf” model. Specifically, in contrast to T cells, the adoptive transfer of NK cells has not led to cytokine release syndrome or GVHD in the allogenic setting. In hematologic malignancies, innate NK cell tumor function is suppressed by cancer directed therapies and the tumor microenvironment and improving NK cell function is vital to the success of cancer treatment. Historically, most clinical trials of adoptive cell therapy have utilized products that are manufactured on a patient-by-patient basis. The high cost of manufacturing per patient delays in care while awaiting a product and variability in the final cell composition make “off-the-shelf” cell therapy highly desirable. Recent optimization of expansion techniques, validation of the safety and scalability of different NK cell sources, and the ability to safely use donor NK cells with minimal HLA matching will allow for true “off-the-shelf” NK cell therapy. To this end, NK cell banks are being developed and will be a timely and cost-effective way to standardize the use of NK cells in cancer therapy. In the future, restoring both the number and function of NK cells throughout a patient’s treatment course may become standard of care by using NK cells from established banks.

The broad applicability of NK cells stems from their ability to kill tumor targets without antigen presentation and pre-clinical and clinical data supporting the use of NK cells across a wide range of malignancies is being published. Particularly with the ubiquitous use of antibody therapy and the importance of NK cells for ADCC, adoptive transfer of NK cells will likely become a universal way to improve the efficacy of this therapeutic modality. In addition to their anti-tumor function, NK cells are important for viral surveillance and exploiting their natural cytotoxicity against viral infected cells may prove to be an additional benefit of the use of NK cells in immunocompromised patients. In vitro data suggests that NK cells may be effective against viral, bacterial, and fungal pathogens [212]. In the setting of haploidentical HSCT for AML, patients treated with NK cells had lower rates of viral reactivation including CMV and BK virus [64]. Clinical trials evaluating the efficacy of adoptive NK cell therapy for treatment of SARS-CoV-2 are underway and if successful may pave the way toward broad use of NK cells for infection indications.

**Fig. 6:**
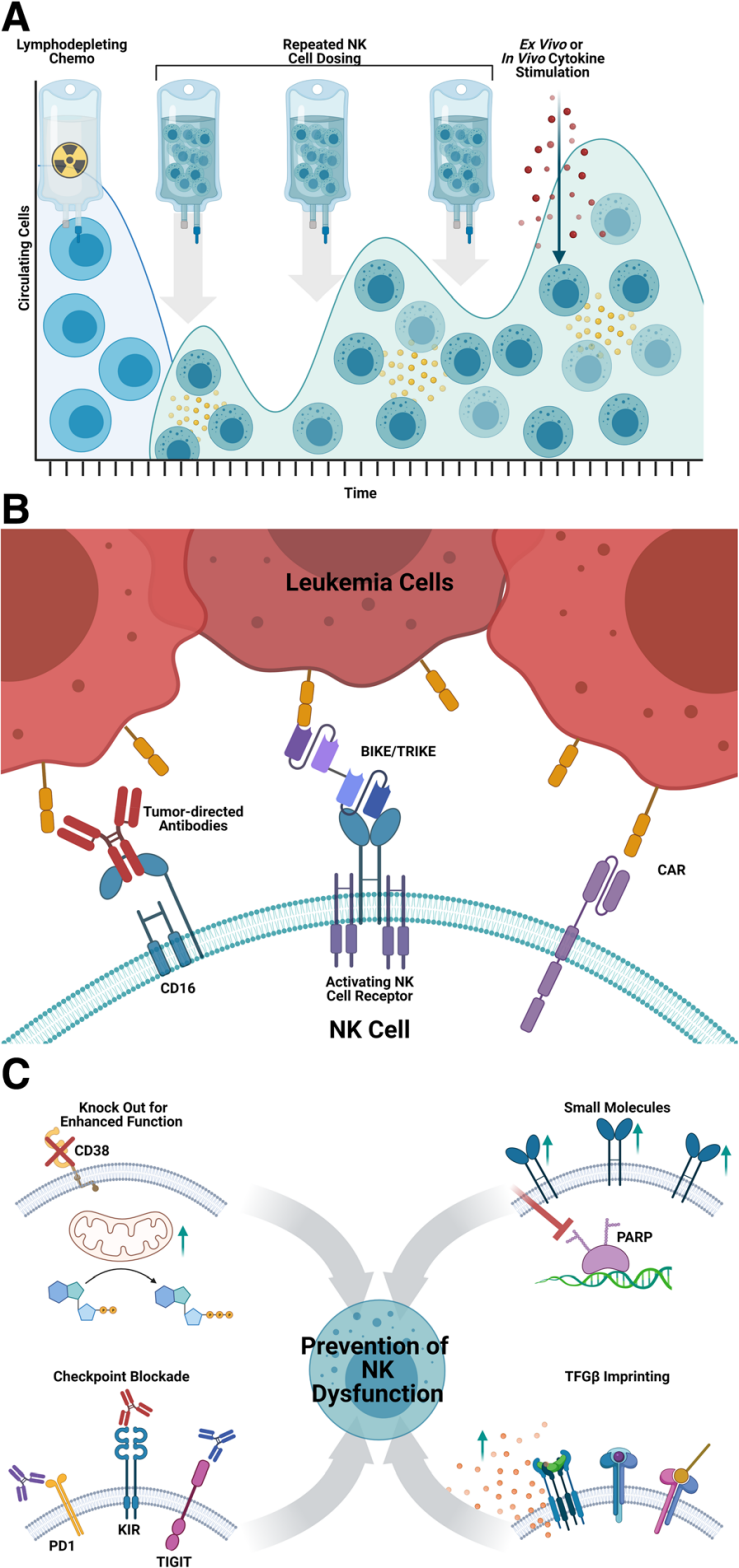
NK cell therapy barriers and solutions. **a** Strategies to improve in vivo persistence of adoptive NK cells include use of lymphodepleting chemotherapy, repeated NK cell doses, exogenous cytokine stimulation, and cytokine secreting “armored” NK cells. **b** Tumor cell immune evasion can be overcome by antibodies directed at the tumor antigen to encourage NK cell ADCC, bispecific engagers interacting with NK activating receptors, or engineered chimeric antigen receptors. **c** Strategies to decrease immune evasion in the tumor microenvironment include (clockwise from the top left) genetic knock out/in of proteins to enhance NK function (ex. CD38 knock out), small molecule inhibitors or immunomodulatory drugs (ex. PARP inhibitors to upregulate NK activating receptors), priming the NK cells ex vivo for preservation of function in vivo (ex. TGFβ imprinting preserving cytolysis and cytokine secretion upon re-exposure to TGF β in the TME), and checkpoint blockade

While there is much optimism for the use of adoptive NK cell therapy in hematologic malignancies, tumor immune evasion remains a barrier to success and strategies to overcome these barriers are actively being investigated (Fig. 6). Specifically, progress made in our ability to genetically engineer NK cells opens up the field to seemingly unlimited therapeutic potential. Novel CAR targets and NK-specific CAR constructs are under development and will be the next major breakthrough for the success of NK cell therapy in hematologic malignancies. With the recent advances discussed in this review, we are on a cusp of an exciting time in the field of adoptive NK cell therapy.

## Data Availability

Not applicable—review manuscript.

## Abbreviations

NK: Natural killer
AML: Acute myeloid leukemia
NHL: Non-Hodgkin lymphoma
HL: Hodgkin lymphoma
GVL: Graft-versus-leukemia
HSCT: Hematopoietic stem cell transplant
NCR: Natural cytotoxicity receptors
KIR: Killer cell immunoglobulin-like receptors
PARP1: Poly-ADP ribose polymerase I
ITIMS: Immunoreceptor tyrosine-based inhibitory motifs
ADCC: Antibody-dependent cellular cytotoxicity
Fc: Crystallizable fragment
BiKE: Bi-specific killer engager
TriKE: Tri-specific killer engager
HIF: Hypoxia inducible factor
IDO: Indoleamine 2,3-dioxygenase
TDO: Tryptophan 2,3-dioxygenase
AhR: Aryl hydrocarbon receptor
TGF- β: Transforming growth factor beta
DNRII: Dominant negative TGF-β receptor
